# The impact of the COVID-19 pandemic on individuals with generalized anxiety disorder: assessing COVID-19 media source exposure and behaviour changes

**DOI:** 10.1186/s12889-022-14510-0

**Published:** 2022-11-11

**Authors:** K. B. Alphonsus, F. Abayateye

**Affiliations:** 1 School of Public Health, University of Saskatchewan, Saskatoon, Canada; 2 Department of Community Health and Epidemiology, College of Medicine, University of Saskatchewan, Saskatoon, Canada

**Keywords:** COVID-19, Generalized anxiety disorder, Mental health, Media, Coping strategies, Canada

## Abstract

**Background:**

The COVID-19 pandemic that has resulted in social distancing, lockdowns, and increase in media posts has taken a toll on the mental health of many people especially those living with Generalized Anxiety Disorder (GAD). The main objective of this study is to understand whether the source of information people use to receive information about COVID-19 and increase or decrease in personal weekly habits during the pandemic were associated with severity of GAD.

**Methods:**

This study was a cross sectional design and was based on data from Canada. The Canadian Perspective Survey Series (CPSS) 4, 2020: Information Sourced Consulted During the Pandemic was used for the study. The outcome variable was severity of GAD. Multivariate logistic regression was carried out using STATA IC 13.

**Results:**

Severity of GAD was significantly associated with being a female, the type of information source used to find out about COVID-19 and change in weekly habits (consuming alcohol, consuming cannabis spending time on the internet and eating junk foods or sweets).

**Conclusion:**

The results indicate that getting information from credible sources about the pandemic, staying connected with family and friends, seeking virtual mental health services, and learning positive coping strategies can help reduce the severity of GAD.

## Background

The social-media age is experiencing its first pandemic – the COVID-19 pandemic. Even though it resembled the 2003 severe acute respiratory syndrome (SARS) epidemic, it is like none other in terms of its rate of spread, number of cases, and deaths recorded. When assessing the length and impact of SARS, it lasted for approximately two years in comparison to COVID-19 which has lingered for many years and has taken more lives than SARS [1]. H1N1 also known as the swine flu took place in 2009 and this was another pandemic that didn’t last that many years [2]. COVID-19 on the other hand was very different in that it came in waves and with many people dying every day, the anxiety of when the pandemic would end or if it would ever end had taken a toll on the mental health of many people globally [3]. The most successful interventions used to slow the spread of the COVID-19 virus throughout the pandemic was social distancing, reduction in social interactions and quarantine measures for individuals who were diagnosed with the illness. The advanced form of these measures was a total lockdown, adopted first by China and Italy and later in other countries. However, the psychological impact of these measures remains debatable.

Human beings are social beings and, as such, love to interact with each other. In times of crisis, an increase in anxiety and uncertainty about current events start to occur and people attempt to resolve their doubts and anxieties through social media to better understand the situation around them [4, 5]. The pandemic has resulted in many countries going into lockdown and this has lead to a period of restricted human interaction, people tend to interact extensively with friends and family through social media sites like Facebook, Twitter, WhatsApp, Instagram and other media outlets to obtain information about the pandemic [6]. People who had been forced to live alone due to isolation or lockdown became very dependent on social media to access information about the increasing number of infections and mortalities [7, 8]. The dependency on social media for information coupled with loneliness due to isolation and lockdown have several repercussions, including increased stress, anxiety, tension, fear, and compulsive obsessions [9–13].

Along with the COVID-19 pandemic, infodemic which is the spread of information through various media platforms also followed [14]. The dissemination of information, particularly false information via social media, has many negative psychological and social consequences for community members [15, 16]. The shock and stress from this misinformation dramatically affected the social and psychological well-being of the public [17, 18].

In the midst of the pandemic one particularly vulnerable group is people with Generalized Anxiety Disorder (GAD). The characteristics of GAD include chronic, extensive worrying with waxing and waning periods and no full remission [19, 20]. These worries mostly centre around uncertainties and varies around many contexts such as work, school, home and social life [19]. In 2012, a survey was carried out among individuals who were 15 years of age an older and it was estimated that 2.5% of Canadians had symptoms related to GAD and 81.2% of individuals reported moderate to severe psychological distress [21]. With media information on COVID-19 creating more tensions and fear, the conditions people with GAD face are likely to be elevated. Mental health problems tend to occur in situations of trauma, less human interaction, and stress. Certain behaviours can be exacerbated by traumatic situations. A study carried out in China demonstrated that COVID-19 anxiety was correlated with severity of problematic smart phone use [22]. Another study indicated that individuals with lifetime psychiatric disorders reported unhealthy behaviours compared to those who did not have psychiatric problems [23]. Individuals with high health anxiety which is most likely present among individuals with GAD are also more likely to suffer from the effects of the COVID-19 pandemic. This health anxiety could increase their anxiety and distress which could influence their behaviour [14]. Not much research has assessed the change in behaviours of people living with GAD especially during the COVID-19 pandemic. The main objective of this study is to understand whether the source of information received about COVID-19 was associated with severity of GAD. As a second objective, we wanted to assess whether increase or decrease in personal weekly habits during COVID-19 were also associated with severity of GAD.

## Methods

### Data source

The data used for this study was from the Canadian Perspective Survey Series (CPSS) 4, 2020: Information Sourced Consulted During the Pandemic. This set of short online cross-sectional surveys started in March 2020 by Statistics Canada. The data collection for series 4 took place between July 20, 2020 to July 26, 2020. The purpose of this survey was used to collect information about the source and quality of COVID-19 on the physical and mental health of individuals living in the 10 Canadian provinces who were 15 years of age or older in timely manner so as to inform policy makers about the state of the nation.

The probability sample was randomly selected from the subset of the Labor Force Survey (LFS) respondents. People living on reserves, other Aboriginal settlements in the provinces, the institutionalized population and households that are in remote areas with very low-density population were excluded from the survey. The LFS survey is from an area frame which is based on a stratified, multi-stage design that uses probability sampling procedures. The LFS used a rotating panel survey design. The participation rate was 23% with a collection response rate of 58.2%. Our analysis used the sampling weights which were created by Statistics Canada. This research used secondary data source; therefore, research ethics board review was exempt.

### Study sample

The initial data set consisted of 4218 cases. The total number of cases used for the study was 3,881 after excluding participants who didn’t answer the questions. Survey weights as recommended by statistics Canada was used for the analysis.

### Variables assessed

The outcome variable of interest was severity of GAD. The categories consisted of no symptoms, minimal symptoms, mild symptoms, moderate symptoms, severe symptoms. The categories were then recoded as follows: 0 = “no symptoms” and 1 = “minimal/mild/moderate/severe symptoms”. The main variable of interest was the main source of information used to find out about COVID-19. The categories were: “news outlet, federal health agency, provincial or territorial health agency, municipal health agency, federal daily announcements, provincial daily announcements, social media, family, friends or colleagues, health professionals, place of employment, other and do not look for information about COVID-19. These categories were then collapsed to ‘news outlet, (federal, provincial or territorial and municipal agency), (federal and provincial daily announcements, provincial daily announcements), social media, (family, friends or colleagues), health professionals, place of employment, other and don’t look for information about COVID-19. Other variables of interest were the change in weekly habits variables where participants were asked “Have your weekly habits changed for any of the following activities? (Consuming alcohol, using tobacco products, consuming cannabis, eating junk food or sweets, watching television, spending time on the internet, playing video games and playing board games). Demographic variables such as sex, age, marital status, respondents’ highest level of education completed and whether there were children under the age of 18 residing in the dwelling were also assessed.

### Analysis

A multivariate logistic regression analysis was carried out to determine whether each of the predictors were associated with severity of GAD. Unadjusted and adjusted odds ratios (ORs) with 95% CIs with p values were computed. Univariate analysis was conducted and variables with *p* < 0.20 were included in the multivariable regression analysis. Manual backward selection was used to build the multivariate model based on (*p* < 0.05). A complete case analysis was conducted in which only variables with complete values were used in the model and missing values were removed. Confounders were tested in the final model and were retained if the addition of that variable changed the coefficients of the main predicting variables by more than 20%. The goodness-of-fit statistics was used to assess model fit. The analysis was performed using STATA IC 13.

## Results

There were 2,548 individuals who reported minimal/mild/moderate/severe symptoms of GAD to while 1,333 individuals reported no symptoms. The total weighted population for the study was 28,889,346. Based on the univariate analysis (Table 1) which is based on weighted data; sex, age, marital status, children under 18 residing in home, consuming alcohol, using tobacco products, consuming cannabis, eating junk food or sweets, watching TV, spending time on the internet, playing video games, playing board games, and the type of source used to find out about COVID-19 were significant at *p* < 0.20. After controlling for education in the final model, sex was significantly associated with severity of GAD. Table 2 shows the results of the final model. The odds of minimal/mild/moderate/severe symptoms versus no symptoms associated with GAD were 1.31 (95%CI 1.02–1.69) times greater for females as opposed to males. Severity of GAD was significantly associated with the type of source used to find out about COVID-19. The odds of individuals having minimal/mild/moderate/severe symptoms of GAD were greater when social media was used to find out the source of information about COVID-19 OR = 2.81 (95% CI 1.31–6.05) compared when individuals did not look for COVID-19 related information. Similarly, when the source of information was from health professionals there was a greater odd of minimal/mild/moderate/severe symptoms being reported by individuals OR = 3.70 (95% CI 1.22–11.18). The odds of minimal/mild/moderate/severe symptoms versus no symptoms associated with GAD were 8.30 (95% CI 1.57–43.88) times greater when other sources were used to gain information about COVID-19 compared to when individuals did not look for information.

**Table 1:** Descriptive statistics and univariate analysis for predictors associated with severity of generalized anxiety disorder. Univariate analysis are based on weighted data set

	Odds Ratio	95%CI	*p-value*	N (unweighted)	N(weighted)
Sex			0.0136*		
Male	ref			1809	14,409,898
Female	1.35	(1.06–1.72)	0.014	2072	14,479,448
Age group			*p* < 0.001*		
15 to 24 years old	ref			167	4,341,744
25 to 34 years old	0.74	(0.36–1.55)	0.431	504	5,123,118
35 to 44 years old	0.49	(0.25–0.94)	0.032	656	4,827,254
45 to 54 years old	0.37	(0.19–0.71)	0.003	647	4,531,533
55 to 64 years old	0.31	(0.16–0.58)	*p* < 0.001	869	4,502,204
65 to 74 years old	0.22	(0.12–0.42)	*p* < 0.001	779	3,990,853
75 years and older	0.17	(0.08–0.36)	*p* < 0.001	259	1,572,641
Marital status			*p* < 0.001*		
Married	0.45	(0.32–0.64)	*p* < 0.001	1989	14,206,215
Living common-law	0.48	(0.31–0.74)	0.001	417	3,246,621
Widowed/Separated/Divorced	0.43	(0.28–0.66)	*p* < 0.001	643	3,080,917
Single/ never married	ref			832	8,355,593
Highest level of education			0.2582		
Less than high school diploma or its equivalent	ref			197	3,586,432
High school diploma or a high school equivalency certificate	1.05	(0.6–1.85)	0.853	726	7,715,138
Trade certificate or diploma	0.62	(0.34–1.13)	0.119	334	2,583,974
College/CEGEP/other non-university certificate or diploma	0.88	(0.52–1.49)	0.631	926	5,881,665
University certificate or diploma below the bachelor’s level	0.75	(0.38–1.52)	0.430	142	729,206
Bachelor’s degree (e.g. B.A. B.Sc. LL.B.)	1.01	(0.6–1.71)	0.956	988	5,688,238
University certificate diploma degree above the BA level	1.01	(0.58–1.76)	0.967	568	2,704,693
Children under 18 residing in home			0.0063*		
No such person resides in the dwelling as of July 20, 2020	ref			2930	18,993,493
Child under 18 on July 20, 2020 resides in dwelling	1.47	(1.11–1.93)	0.006	951	9,895,853
Change in weekly habits					
Consuming alcohol			*p* < 0.001*		
Increased	2.98	(1.9–4.68)	*p* < 0.001	600	4,756,656
Decreased	3.07	(1.9–4.97)	*p* < 0.001	339	3,162,146
No change	ref			2942	20,970,544
Using tobacco products			0.0027*		
Increased	3.02	(1.61–5.7)	0.001	145	1,194,921
Decreased	1.28	(0.4–4.1)	0.677	65	517,743
No change	ref			3671	27,176,682
Consuming cannabis			*p* < 0.001*		
Increased	9.89	(5.23–18.73)	*p* < 0.001	199	1,760,207
Decreased	1.18	(0.41–3.4)	0.762	68	530,644
No change	ref			3614	26,598,495
Eating junk food or sweets			*p* < 0.001*		
Increased	4.39	(3.1–6.22)	*p* < 0.001	992	7,735,999
Decreased	2.65	(1.86–3.76)	*p* < 0.001	508	4,427,447
No change	ref			2381	16,725,900
Watching TV			*p* < 0.001*		
Increased	2.35	(1.8–3.07)	*p* < 0.001	1670	13,318,441
Decreased	1.82	(1.22–2.71)	0.003	332	2,365,316
No change	ref			1879	13,205,589
Spending time on the internet			*p* < 0.001*		
Increased	2.74	(2.13–3.54)	*p* < 0.001	1953	16,402,796
Decreased	1.89	(1.1–3.25)	0.022	148	911,834
No change	ref			1780	11,574,716
Playing video games			*p* < 0.001*		
Increased	2.67	(1.8–3.96)	*p* < 0.001	531	5,592,581
Decreased	2.26	(1.22–4.18)	0.009	111	988,592
No change	ref			3239	22,308,174
Playing board games			*p* < 0.001*		
Increased	2.10	(1.52–2.9)	*p* < 0.001	576	4,730,146
Decreased	1.82	(0.94–3.53)	0.077	114	852,186
No change	ref			1191	23,307,014
Main source of information to find out about COVID-19			0.0013*		
News outlet	1.87	(0.97–3.61)	0.064	1935	14,471,158
Federal provincial territorial and municipal health agency	2.37	(1.16–4.82)	0.018	579	3,774,693
Federal and provincial daily announcements	1.57	(0.76–3.23)	0.225	573	3,691,356
Social media	3.46	(1.67–7.16)	0.001	326	2,845,776
Family friends or colleagues	1.72	(0.63–4.75)	0.293	137	1,500,877
Health professionals	4.07	(1.43–11.6)	0.009	72	649,856
Place of employment	2.10	(0.83–5.33)	0.118	112	772,999
Other	9.72	(2.01–47.08)	0.005	33	399,664
Don’t look for information	ref			114	782,966

^*^Significance is based on *p* < 0.20

**Table 2:** Multivariate logistic regression model assessing the association between severity of generalized anxiety disorder and variables of interest based on weighted data

	OR	95%CI	*p-value*
Sex			0.032*
Male	Ref		
Female	1.31	(1.02–1.69)	0.032
Age group			0.077
15 to 24 years old	ref		
25 to 34 years old	0.89	(0.42–1.93)	0.777
35 to 44 years old	0.68	(0.33–1.40)	0.300
45 to 54 years old	0.60	(0.29–1.24)	0.168
55 to 64 years old	0.54	(0.26–1.13)	0.102
65 to 74 years old	0.37	(0.17–0.82)	0.014
75 years and older	0.37	(0.16–0.87)	0.023
Marital status			0.796
Married	0.91	(0.61–1.36)	0.655
Living common-law	0.81	(0.52–1.26)	0.344
Widowed/Separated/Divorced	0.96	(0.60–1.53)	0.867
Single never married	ref		
Highest level of education			0.751
Less than high school diploma or its equivalent	ref		
High school diploma or a high school equivalency	1.05	(0.57–1.94)	0.865
Trade certificate or diploma	0.76	(0.4–1.46)	0.406
College/CEGEP/other non-university certificate or diploma	0.97	(0.53–1.79)	0.929
University certificate or diploma below the bachelor’s level	0.96	(0.44–2.08)	0.911
Bachelor’s degree (e.g. B.A. B.Sc. LL.B.)	1.08	(0.58–2.00)	0.813
University certificate diploma degree above the BA level	1.09	(0.57–2.09)	0.788
Children under 18 residing in home			0.502
No such person resides in the dwelling as of July 20, 2020	ref		
Child under 18 on July 20 2020 resides in dwelling	0.88	(0.61–1.28)	0.503
Change in weekly habits			0.0002*
Consuming alcohol			
Increased	1.92	(1.23–3.00)	0.004
Decreased	2.10	(1.35–3.26)	0.001
No change	ref		
Using tobacco products			0.297
Increased	1.42	(0.71–2.86)	0.322
Decreased	0.53	(0.19–1.51)	0.233
No change	ref		
Consuming cannabis			*p* < 0.001*
Increased	4.59	(2.31–9.11)	*p* < 0.001
Decreased	0.52	(0.20–1.35)	0.18
No change	ref		
Eating junk food or sweets			*p* < 0.001*
Increased	2.46	(1.70–3.56)	*p* < 0.001
Decreased	1.85	(1.27–2.68)	0.001
No change	ref		
Watching tv			0.2181
Increased	1.31	(0.96–1.79)	0.086
Decreased	1.05	(0.68–1.62)	0.834
No change	ref		
Spending time on the internet			0.0061*
Increased	1.58	(1.19–2.09)	0.002
Decreased	1.35	(0.70–2.57)	0.368
No change	ref		
Playing video games			0.360
Increased	1.24	(0.81–1.90)	0.320
Decreased	1.60	(0.73–3.51)	0.241
No change	ref		
Playing board games			0.463
Increased	1.25	(0.88–1.78)	0.215
Decreased	1.07	(0.47–2.41)	0.872
No change	ref		
Main source of information to find out about COVID-19			0.044*
News outlet	1.90	(0.95–3.80)	0.071
Federal provincial territorial and municipal health agency	1.94	(0.92–4.12)	0.083
Federal and provincial daily announcements	1.57	(0.72–3.43)	0.253
Social media	2.81	(1.31–6.05)	0.008
Family friends or colleagues	1.12	(0.35–3.58)	0.854
Health professionals	3.70	(1.22–11.18)	0.021
Place of employment	1.69	(0.58–4.92)	0.333
Other	8.30	(1.57–43.88)	0.013
Don’t look for information	ref		

^*^Significance is based on *p* < 0.05

Similarly, the odds of having minimal/mild/moderate/severe symptoms of GAD were greater when there was a decrease in consuming alcohol OR = 2.10 (95% CI 1.35–3.26). The odds of minimal/mild/moderate/severe symptoms were also greater for when respondents reported an increase in alcohol consumption, however the effect was less OR = 1.92 (95% CI 1.23–3.00) compared to when respondents reported decreased consumption. When assessing cannabis consumption, the odds of minimal/mild/moderate/severe symptoms versus no symptoms associated with GAD were 4.59 (95% CI 2.31–3.56) times greater when the change in weekly cannabis consumption increased compared to when there was no change in the consumption behaviour. The odds of having minimal/mild/moderate/severe symptoms of GAD were 2.46 (95%1.70–3.56) times greater when there was an increase in the change in weekly habit of eating junk foods or sweets compared to when there was no change. The odds were also similar when there was a decrease in the consumption of eating junk food or sweets but slightly less than when there was an increase OR = 1.85 (95%CI 1.27–2.68). The odds of minimal/mild/moderate/severe symptoms of GAD were 1.58 (95% CI 1.19–2.09) times greater when the amount of time spent on the internet was increased as opposed to when there was no change in the weekly habits.

## Discussion

### Main findings of this study

The main objective of this study was to assess whether sources of information about COVID-19 was associated with severity of GAD. Out of all the sources of information used to gain information about COVID-19, individuals who used social media had an increased odds of having minimal/mild/moderate/severe symptoms of GAD. In general, social media tends to contain more misinformation being circulated about the pandemic [24]. This can provoke fear and for people with a mental health problem such as anxiety could heighten their existing symptoms even though the information may or may not be true. Several studies point to increased mental health problems as a result of spending too much time on social media to find out about COVID-19 [5, 8, 25]. The results of the study also indicated that increased time spent on the internet was associated with minimal/mild/moderate/severe symptoms of GAD. Anxious individuals will be more inclined to seek out information about how to protect themselves from COVID-19. This is good and bad since, seeking out information can help you better prepare yourself from COVID-19 but it can also increase anxiety depending on the available information on social media [26–28]. This can lead to a vicious cycle which is detrimental for people living with this disorder. Other ways social media could increase anxiety is through the bombardment of pages that are being liked, shared and circulated numerous times. Even though a person may not actively seek out COVID-19 related information, pages with stories pop up often and this can trigger anxiety among people with underlying conditions.

The pandemic has caused a great deal of stress among health professionals and their goal is to protect the public and prevent more cases from coming into hospitals. Health care professionals have faced a great amount of stress during the pandemic with having to work over time and this has to lead to an increase in suicide rates [29]. Therefore, any information that health professionals provide to the public would be taken seriously since they see firsthand what is happening to patients with COVID-19 and the mental health problems that has occurred because of the pandemic [30]. This could be the reason for the increased odds of GAD that is present in our findings. Using other sources of information about COVID-19 were associated with increased odds of minimal/mild/moderate/severe symptoms of GAD among respondents compared to any of the other categories. Conspiracy theories are another factor in spreading false information about COVID-19 and with new podcasts these theories can spread around the world [31]. Such theories include the idea that COVID-19 was part of a global conspiracy theory, 5G networks were helping in spread of COVID-19 or the pandemic was part of a biological warfare to name a few [32]. One of the best ways to reduce misinformation is to share stories on the news about the amount of misinformation online and redirect the public to reputable sources of information.

When assessing demographic factors, females were more likely than males to experience a minimum to severe symptoms of GAD. During the pandemic, Statistics Canada reports that there were gender differences in mental health status among the Canadian population [33]. Female participants were more likely than males to report moderate or severe GAD. The reason for this difference could be because of the amount of time females spend taking care of their children and doing household work [34]. Since many parts of the provinces were in lockdown throughout year, lack of social interaction combined with the stress of household work could worsen symptoms of anxiety disorders [35]. The stress of COVID-19 has been shown to exacerbate many underlying mental health conditions such as GAD and depression [36].

### What is already known on this topic

Research evidence suggests that during disease outbreaks (epidemic or pandemic), mental health issues such as anxiety increases [37–39]. Also, pre-existing anxiety disorder, existing health anxiety, and other mental health disorders can increase anxiety levels during these periods, observed with the COVID-19 pandemic [40]. With COVID-19 restrictions taking place during each wave of the pandemic, there has also been an increase in the amount of time people spend in isolation which is a factor for the rise in mental health problems globally [41]. The Centers for Disease Control and Prevention has listed some healthy means of coping with stress and anxiety during the COVID-19 pandemic [42]. These include meditation, healthy eating, regular exercise, sufficient sleep, avoidance of alcohol, tobacco and other substance, and adherence to routine preventive measures [42]. Unfortunately, research has shown that most people who undergo stress and anxiety during the COVID-19 pandemic engage in the complete opposite of these suggestions.

### What this study adds

Our study also indicated that change in weekly habits such as increased consumption of alcohol, cannabis and eating junk foods were also associated with minimal/mild/moderate/severe symptoms of GAD. During times of hardship, coping mechanisms are vastly used by individuals. This was consistent with other findings carried out during the pandemic where in another study, one third of Canadians with anxiety and depression also reported to have increased their alcohol and cannabis intake [43]. Other studies indicate that increased cannabis use was associated with younger age groups and being somewhat worried about the pandemics impact on personal finance [44]. Self-isolation and depression were found to be a significant contributor to cannabis use [45]. More alcohol consumption was also found to be significantly greater during the pandemic than before [46, 47]. Consuming alcohol was associated with younger age groups, having more children at home, being a non-health care worker and being unemployed [46]. Adults who had pre-existing mental illness such as anxiety and depression were also more likely to increase their alcohol use during the COVID-19 pandemic [48].

Individuals may not have access to mental health services and may self-medicate with alcohol, cannabis and eating junk food. A study in the UK, reported that the increased consumption of foods during lockdown were part of higher maladaptive coping strategies [49]. Maladaptive coping strategies may lead to addiction in the future. Learning to reduce the stress through family support and engaging in activities that promote positive moods such as cooking, video chats, facetime, Netflix movie parties can help people stay connected with others and feel less isolated [50, 51]. Access to virtual mental health services is of great importance during the pandemic since more people will be able to benefit from virtual services even if they are not able to leave the house. Asynchronous virtual mental health resources for COVID-19 exist in Canada, however not many people are accessing them. Promoting these services on social media or through news channels would reach a wider range of people [52]. Clinicians should also promote the services so that patients are aware of online mental health support services and can reach out in immediate distress.

### Limitations of this study

This study is based on cross-sectional secondary source data that was collected during the early wave of the COVID-19 pandemic, therefore a cause an effect relationship cannot be determined. In addition to this, behavioural patterns may have changed when restrictions were lifted in some provinces over time that could have led to a decrease in unhealthy habits which could not be captured in this study. This research used weights to produce estimates based on Statistics Canada recommendations. The survey used a bootstrap resampling method but was not available in the public use file. Therefore, the variance for the odds ratios could not be adjusted which may results in underestimation of the variability.

## Conclusions

Our study indicated that the type of platform used to gain information about COVID-19 was associated with severity of GAD. Reasons for increased anxiety could be due to the amount of misinformation that circulates on the internet. The public needs to be aware that if the news about the pandemic is not from a reputable source, it should not be a cause of concern. The pandemic has left a lot of people feeling anxious, even individuals without pre-existing mental health problems. To reduce anxiety, connecting with family and friends through video calls and learning to keep positive through various activities can help with having a positive outlook on life. Mental health organizations need to promote online virtual programs for people so that they can get the help they need even if they are at home. More positive messages about the pandemic and sense of hope also needs to be shown on social media, television and other types of media platforms so that people will feel that they will be okay and that there is hope for the future.

## Data Availability

The datasets generated and/or analysed during the current study are available through Statistics Canada, https://www150.statcan.gc.ca/n1/daily-quotidien/200817/dq200817b-eng.htm.
